# Artificial intelligence in emergency trauma perioperative management: applications, performance benchmarks, and translation barriers

**DOI:** 10.3389/fsurg.2026.1970304

**Published:** 2026-10-26

**Authors:** Lu Yang, Chunping Li, Xuren Wang, Yan Zeng, Hua Zhang

**Affiliations:** 1 Department of Emergency Medicine, Hainan General Hospital, Affiliated Hainan Hospital of Hainan Medical University, Haikou, Hainan, China; 2 Nursing Department, Changhai Hospital, Naval Medical University, Shanghai, China; 3 Nursing Department, Hainan Health Vocational College, Haikou, Hainan, China; 4 School of Nursing, Hainan Medical University, Haikou, Hainan, China

**Keywords:** diagnostic imaging, emergency trauma, machine learning, multimodal data fusion, predictive analytics

## Abstract

This review examines the current status of artificial intelligence (AI) applications in the perioperative management of emergency trauma. Published studies suggest performance advantages of AI over traditional methods in prehospital triage, radiographic injury detection, massive transfusion prediction, and early warning of postoperative complications, although findings vary across study designs and validation settings. However, actual adoption in trauma care remains limited due to data heterogeneity, performance degradation in external validation, workflow integration challenges, lack of interpretability, and a scarcity of prospective interventional evidence. This review further argues that methodologically inflated benchmark performance constitutes an underrecognized barrier to adoption in its own right, and that bridging the implementation gap will likely require a shift from area under the receiver operating characteristic curve (AUC)-centered reporting toward reporting sensitivity at prespecified clinical thresholds and evaluating patient outcomes in prospective interventional trials.

## 1 Background

Emergency trauma represents a leading global cause of mortality and disability, imposing unique clinical demands characterized by time-critical decision-making and complex pathophysiological cascades (1–3). Traditional perioperative management approaches face substantial limitations when confronted with polytrauma presentations, where reliance on clinical experience and constrained real-time data processing capacity can result in diagnostic delays exceeding the critical golden hour window, directly contributing to increased mortality rates (4, 5). The advent of artificial intelligence (AI), encompassing its core methodologies of machine learning (ML) and deep learning (DL), has introduced computational frameworks capable of processing multimodal clinical data at scales and speeds unattainable through conventional human-based analysis (6, 7). ML algorithms employ iterative pattern recognition from structured clinical datasets to generate predictive models, while DL architectures, particularly convolutional neural networks (CNNs), demonstrate capacity for automated feature extraction from unstructured data including radiological imaging and free-text clinical documentation (8).

Recent clinical investigations have documented quantifiable performance improvements attributable to AI implementation across multiple domains of trauma care (9). In prehospital triage applications, ML-based prediction models have reported values ranging from 0.75 to 0.93 for the area under the receiver operating characteristic curve (AUC), with demonstrated capacity to reduce undertriage rates below 10% compared to conventional guideline-based protocols (10, 11). In radiological diagnostics, commercial deep learning algorithms for intracranial hemorrhage detection on noncontrast computed tomography have demonstrated overall accuracy ranging from 93.0% to 96.6% and specificity exceeding 93% in real-world emergency settings; however, prospective evaluation indicates that AI triage systems may not consistently improve upon experienced radiologists' baseline diagnostic performance (12–15). Mortality prediction models utilizing AI have reported AUC values as high as 0.9974 [retrospective, multicenter, *n* = 6,536,306, no independent external validation] (16); however, as discussed later in this review, such extreme performance metrics in trauma populations often signal methodological artifacts rather than true clinical utility. Beyond diagnostic applications, AI-driven intelligent monitoring systems now integrate continuous physiological data streams from wearable devices, enabling detection of subtle hemodynamic deterioration patterns indicative of evolving complications such as coagulopathy and compartment syndrome (8, 17). In surgical decision support, AI algorithms have been applied to predict the necessity for major surgical intervention and massive transfusion protocol activation, potentially optimizing resource allocation in trauma centers (10, 18).

Despite these documented performance metrics, clinical translation of AI technologies remains substantially limited, with adoption in emergency, critical, and perioperative medicine described as limited, fragmented, and geographically uneven across high-resource healthcare systems (19). This implementation gap stems from multiple interconnected challenges. Data heterogeneity across institutions may produce differential model performance when models are deployed beyond their training environments, with some studies reporting reductions of approximately 3% in prehospital mortality prediction across international cohorts (20), while others show greater degradation in geographically distinct populations (21). The algorithmic opacity inherent to deep learning architectures has generated persistent concerns regarding clinical interpretability, with survey and consensus evidence identifying limited clinician acceptance of, and difficulties interacting with, opaque AI outputs as major limitations for deployment in emergency care (22). Furthermore, ethical and regulatory frameworks have failed to establish clear liability boundaries for AI-assisted clinical decisions, while data governance structures inadequately address privacy protection requirements under regulations such as the Health Insurance Portability and Accountability Act (HIPAA) and the General Data Protection Regulation (GDPR) (23, 24). Notably, prospective validation through randomized controlled trials remains scarce, with current evidence predominantly derived from retrospective, single-center cohort studies that introduce selection bias and limit generalizability (12, 25).

This narrative review critically examines the current state of AI integration in emergency trauma perioperative management. We synthesize evidence regarding AI applications across the continuum of trauma care, from prehospital triage through perioperative monitoring, while evaluating the clinical performance metrics that distinguish effective implementations from those demonstrating methodological limitations. The review addresses three central questions: First, which AI applications have demonstrated sufficient clinical validation to warrant integration into trauma workflows? Second, what technical, clinical, and regulatory barriers currently impede broader adoption? Third, what strategic priorities should guide future development to bridge the existing gap between algorithmic potential and clinical implementation? By providing a structured analysis of these dimensions, this review aims to inform clinicians, researchers, and policymakers regarding evidence-based pathways for advancing AI-assisted trauma care.

### 1.1 Literature search strategy

A structured literature search was conducted in the PubMed, Web of Science, and Scopus databases for articles published from January 2016 to August 2026, with the final search conducted on August 10, 2026. The complete search strings applied to each database were as follows. For PubMed: (“machine learning”[Title/Abstract] OR “deep learning”[Title/Abstract] OR “artificial intelligence”[Title/Abstract] OR “neural network*”[Title/Abstract]) AND (“trauma*”[Title/Abstract] OR “injur*”[Title/Abstract]) AND (“hemorrhage”[Title/Abstract] OR “haemorrhage”[Title/Abstract] OR “bleeding”[Title/Abstract] OR “resuscitation”[Title/Abstract] OR “massive transfusion”[Title/Abstract] OR “shock”[Title/Abstract]). For Web of Science: TOPIC: (“artificial intelligence” OR “machine learning” OR “deep learning” OR “neural network”) AND TOPIC: (“trauma” OR “emergency medicine” OR “triage” OR “prehospital” OR “hemorrhage” OR “resuscitation” OR “perioperative care” OR “clinical decision support”). For Scopus: TITLE-ABS-KEY (“artificial intelligence” OR “machine learning” OR “deep learning” OR “neural network”) AND TITLE-ABS-KEY (“trauma” OR “emergency medicine” OR “triage” OR “prehospital”) AND TITLE-ABS-KEY (“hemorrhage” OR “resuscitation” OR “perioperative care” OR “clinical decision support”). Additional hand searches of reference lists from included studies and relevant systematic reviews were performed to identify further publications. Inclusion criteria comprised original research articles, systematic reviews, and meta-analyses reporting AI or ML applications in emergency trauma care, including prehospital triage, diagnostic imaging, perioperative resuscitation and decision support, and postoperative monitoring. We excluded non-English publications, conference abstracts without available full texts, editorials without original data, and studies focused exclusively on non-trauma emergency conditions. Two reviewers independently screened titles and abstracts, followed by full-text evaluation of potentially eligible articles. Disagreements were resolved through consensus discussion. The database search retrieved 2,550 records (PubMed, *n* = 720; Web of Science, *n* = 1,324; Scopus, *n* = 506). An additional 5 records were identified through manual searching of reference lists from included studies and relevant systematic reviews, yielding a total of 2,555 records. After removal of 655 duplicates, 1,900 records underwent title and abstract screening, of which 1,710 were excluded. Full-text review was performed for 190 articles, of which 116 were excluded because they were not relevant to emergency trauma care (*n* = 71), provided insufficient detail on the AI or ML application to permit data extraction (*n* = 28), or reported duplicate data from an already included study (*n* = 17), leaving 74 articles that were included in this narrative synthesis (Figure 1). Data extraction included study design, sample size, AI methodology, performance metrics (AUC, sensitivity, specificity, F1 score), validation approach (internal vs. external), and clinical translation status. The risk of bias in reported model-performance evaluations was examined using the four domains of the Prediction model Risk Of Bias ASsessment Tool (PROBAST): participants, predictors, outcome, and analysis. We interpreted these domains in light of the distinction between model development and performance evaluation emphasized by PROBAST + AI (26), while retaining domain-level judgments under the original PROBAST framework. Eligible evaluations developed and assessed a clinical prediction or classification model, or independently assessed the performance of an existing model, and reported discrimination or diagnostic accuracy in a defined clinical cohort. For existing commercial image classifiers, the appraisal also examined the independence and masking of the diagnostic reference standard, differential verification, patient selection, and repeat examinations. In addition to the 12 model-performance evaluations appraised with PROBAST (Supplementary Table S1), four studies that evaluated the diagnostic accuracy of pre-trained commercial AI classifiers against a reference standard were independently assessed using QUADAS-2 (Quality Assessment of Diagnostic Accuracy Studies, version 2) (13–15, 27), which examines patient selection, index test, reference standard, and flow and timing domains. These four QUADAS-2 assessments are presented in Supplementary Table S2. Internal validation was defined as evaluation within the development data source (including split-sample testing, resampling and cross-validation); temporal external validation used a later non-overlapping period, and geographic external validation used independent institutions or countries. Independent testing of a previously developed commercial imaging classifier was labelled external clinical performance evaluation, not local model development. Risk of bias was separated from applicability to emergency trauma care; missing methodological details were assigned Unclear rather than being presumed Low. Secondary syntheses, editorials, models without clinical performance evaluation and process-only workflow studies were not scored. Of the 74 articles included in the narrative synthesis, 12 model-performance evaluations met the criteria for PROBAST risk-of-bias assessment and are appraised in Supplementary Table S1. Among these 12, four studies that specifically evaluated pre-trained commercial diagnostic imaging classifiers were additionally assessed using QUADAS-2, with results presented in Supplementary Table S2 (13–15, 27). This review employed a structured narrative synthesis based on a systematic literature search. Although the breadth and heterogeneity of included AI applications across diverse clinical contexts precluded formal meta-analysis, a structured and reproducible search strategy, predefined inclusion and exclusion criteria, and dual-reviewer independent screening were employed to minimize selection bias and enhance transparency.

**Figure 1:**
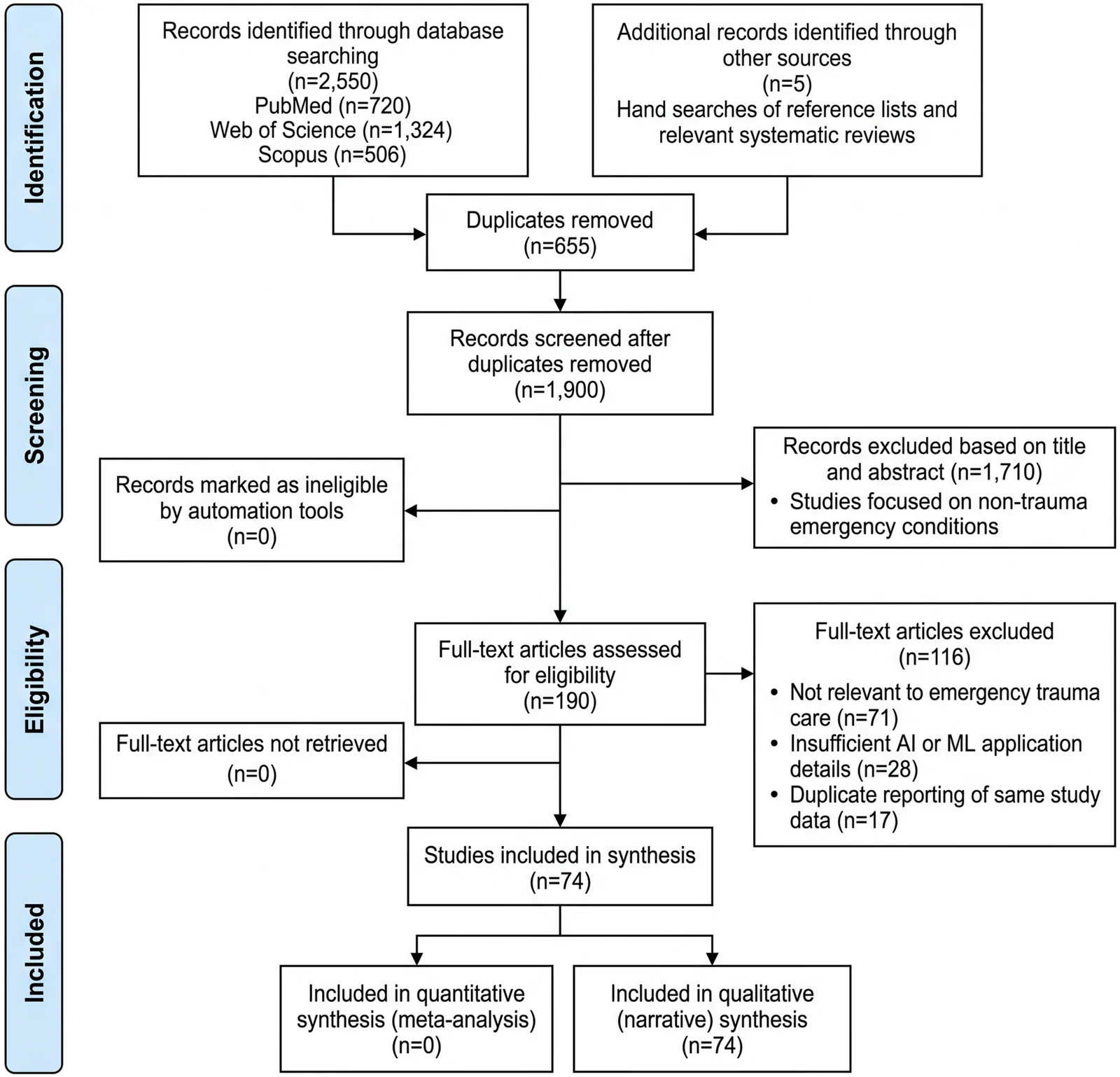
PRISMA flow diagram of study selection process. This figure was created using Microsoft PowerPoint.

## 2 Clinical demands in perioperative management of emergency trauma patients

### 2.1 Pathophysiological characteristics

Emergency trauma initiates complex pathophysiological cascades that fundamentally distinguish perioperative management requirements from elective surgical care. Trauma-induced coagulopathy, a life-threatening condition affecting 25%–35% of severely injured patients, develops through mechanisms including endothelial glycocalyx degradation, activated protein C upregulation, and platelet dysfunction, resulting in bleeding that accounts for 30%–40% of trauma mortality (28, 29). These coagulopathic derangements generate massive volumes of high-dimensional temporal data, including serial coagulation profiles, thromboelastography parameters, and fibrinolytic markers that exceed the processing capacity of traditional manual interpretation systems (29, 30).

Concurrent immune-inflammatory dysregulation further compounds management complexity. Following major trauma, patients experience complex immune dysregulation that was traditionally conceptualized as a biphasic sequence of systemic inflammatory response syndrome followed by compensatory anti-inflammatory response syndrome; however, emerging evidence indicates these processes may occur concurrently rather than sequentially, with approximately 30%–43% of severely injured patients developing sepsis following admission (28, 31). This immunologic volatility necessitates continuous reassessment of infection risk, yet conventional clinical scoring systems demonstrate limited predictive accuracy, with area under the curve values typically ranging from 0.60 to 0.75 for early sepsis detection (31).

The lethal triad of hypothermia, acidosis, and coagulopathy represents a well-established pathophysiological framework, though recent evidence has expanded this concept to include hypocalcemia as a fourth critical component referred to as the “diamond of death”. Hypocalcemia, present in up to 70% of massively transfused trauma patients, impairs coagulation cascade function and myocardial contractility, yet remains inadequately addressed in traditional resuscitation protocols (4). Population-specific vulnerabilities further stratify clinical needs. Geriatric trauma patients, comprising an increasing proportion of admissions, exhibit attenuated physiological responses to injury secondary to reduced functional reserve and comorbidity burden. Standard triage tools demonstrate undertriage rates exceeding 20% in patients over 65 years, as conventional vital sign thresholds fail to capture age-related compensatory limitations (32, 33). Similarly, emerging biomarkers such as cell-free hemoglobin levels, which increase within 1 h after severe injury and correlate with subsequent organ dysfunction, offer predictive capacity but lack integration into real-time clinical workflows due to measurement complexity and interpretation challenges (34).

### 2.2 Time-sensitivity challenges

Trauma care operates within critically constrained temporal windows where diagnostic and therapeutic delays directly correlate with mortality. The golden hour principle, empirically established through military and civilian trauma systems, posits that definitive hemorrhage control within 60 min of injury optimizes survival outcomes. However, contemporary data demonstrate that this window may be substantially shorter for specific injury patterns, with time-critical interventions for severe hemorrhage ideally initiated within the first resuscitation phase (35). Within this compressed timeframe, clinicians must synthesize multimodal unstructured data including complex radiological images, free-text prehospital reports, and dynamic vital sign trends to formulate treatment decisions (18, 36).

Current triage systems inadequately address this time-critical complexity. Traditional graded triage approaches, relying on anatomic injury scoring and physiologic parameters, demonstrate undertriage rates of 10%–35% for patients requiring major trauma center resources, directly resulting in delayed definitive care and increased mortality (5, 37, 38). Conversely, overtriage rates of 25%–50% in many systems result in inefficient resource utilization and potential harm through unnecessary rapid transport (10, 39). The challenge intensifies in mass casualty scenarios where conventional individual assessment approaches become operationally infeasible due to overwhelming patient volumes relative to available providers (11).

Radiological interpretation presents particular time-sensitivity constraints. While computed tomography scanning has become the diagnostic standard for polytrauma evaluation, imaging volume demands and multi-system complexity create critical bottlenecks in interpretation workflow, increasing the risk of delayed reporting and missed findings pending formal radiologist review (36).

Resuscitation strategy selection faces similar temporal pressures. Massive transfusion protocols, while improving outcomes through early hemostatic resuscitation, require dynamic adjustment based on evolving coagulopathy profiles that change minute-to-minute during active hemorrhage (40, 41). Traditional static protocols employing fixed plasma-to-red blood cell ratios fail to accommodate this physiologic variability, potentially contributing to both under-resuscitation and transfusion-related complications. Real-time assessment of fibrinogen levels, platelet function, and fibrinolytic activity through viscoelastic testing provides mechanistic guidance, yet interpretation requires specialized expertise often unavailable during overnight and weekend hours (30, 40).

### 2.3 Multidisciplinary collaboration demands

Effective trauma perioperative management necessitates coordinated input from multiple specialties including emergency medicine, trauma surgery, anesthesiology, interventional radiology, orthopedic surgery, neurosurgery, and critical care medicine, yet traditional verbal handoffs between prehospital providers and emergency teams frequently omit critical details regarding mechanism of injury, prehospital interventions, and treatment response, undermining the collective decision-making upon which optimal outcomes depend (2).

Surgical decision-making exemplifies multidisciplinary complexity. Determination of operative vs. non-operative management for solid organ injuries requires integration of radiological findings, hemodynamic trajectory, coagulation status, and physiologic reserve, typically incorporating input from trauma surgeons, interventional radiologists, and intensivists. This collaborative assessment process introduces substantial time costs in time-critical scenarios, with decision quality dependent on the availability and experience of multiple providers (30).

Perioperative anesthetic management demands real-time synthesis of evolving patient physiology with surgical requirements. Trauma patients frequently present with full stomachs, hemodynamic instability, and uncertain injury burden, necessitating anesthetic techniques that balance aspiration risk, cardiovascular support, and neurologic monitoring. Traditional anesthetic decision algorithms, developed for elective surgical populations, demonstrate limited applicability, requiring individualized approaches based on mechanism of injury, anatomic injury pattern, and physiologic derangement severity (3).

Postoperative care transitions present additional coordination challenges. Transfer of critically injured patients from the operating room to the intensive care unit requires handoff of complex physiologic data, ongoing resuscitation requirements, and planned interventions across nursing and physician teams. Communication failures during these transitions represent a recognized source of adverse events, including delayed recognition of ongoing hemorrhage, medication errors, and ventilator management complications (1).

Beyond acute care phases, psychological and rehabilitation needs introduce long-term multidisciplinary requirements. Post-traumatic stress disorder represents a prevalent complication among trauma survivors, substantially impacting quality of life and functional recovery trajectories (42). Traditional trauma systems focus primarily on acute physiologic stabilization, with limited integration of mental health screening and intervention. Stratified collaborative care models incorporating early psychological assessment, graduated rehabilitation programming, and social support coordination demonstrate improved outcomes, yet implementation across trauma centers remains inconsistent (43). The complex interplay between the pathophysiological characteristics of emergency trauma and the inherent bottlenecks of traditional management strategies is summarized in Figure 2.

**Figure 2:**
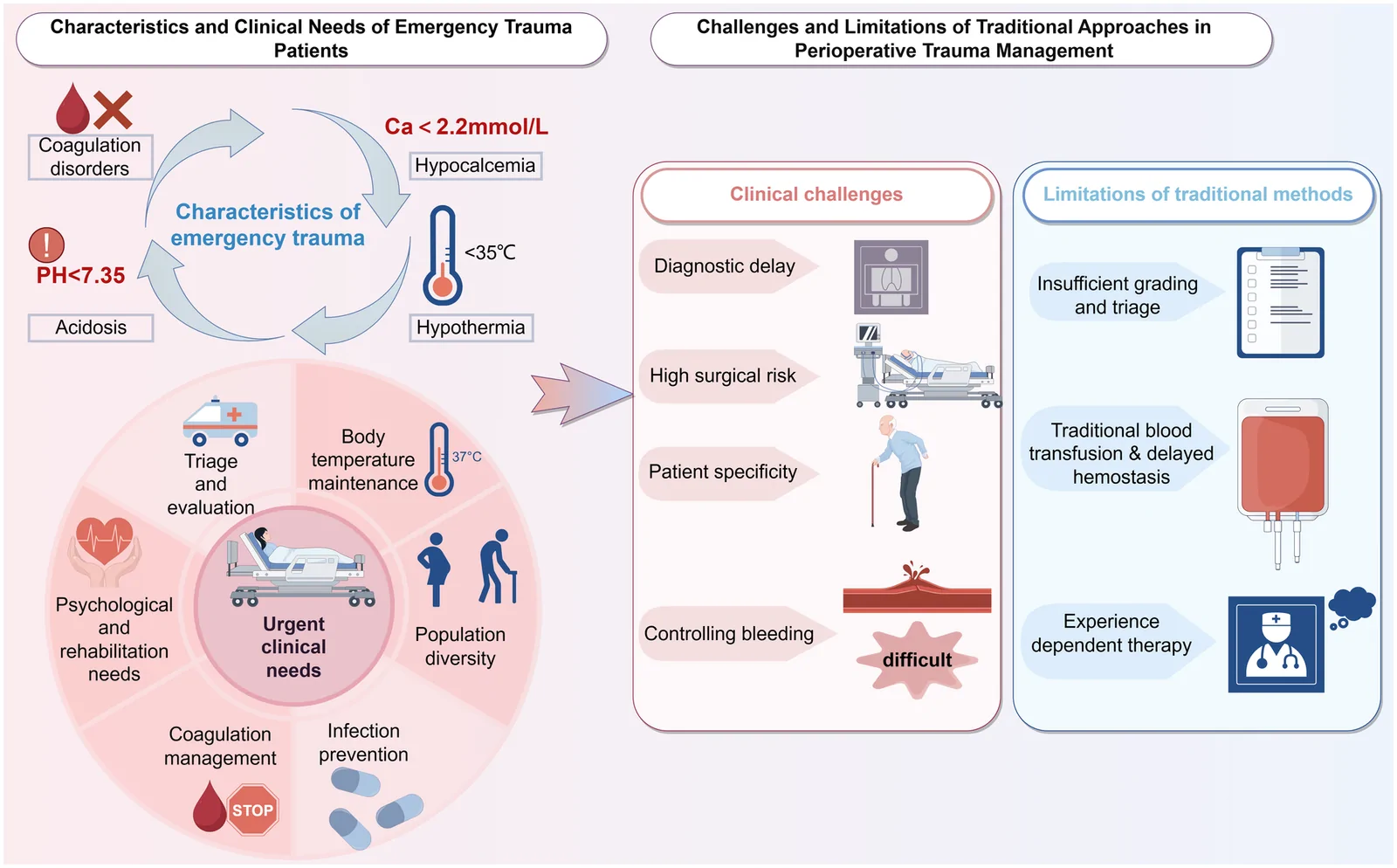
Characteristics and clinical needs of emergency trauma patients vs. limitations of traditional perioperative management. The left panel illustrates the pathophysiological “diamond of death” (acidosis, hypothermia, coagulopathy, and hypocalcemia) driving urgent clinical needs such as rapid triage, temperature maintenance, and coagulation control. The right panel highlights key challenges in traditional approaches, including diagnostic delays, high surgical risks, patient specificity issues, and reliance on experience-dependent therapy, which collectively underscore the necessity for intelligent decision-support systems. This figure was created by the authors using Figdraw (https://www.figdraw.com; ID: WATYO6b55b), an online scientific illustration platform.

## 3 AI applications in emergency trauma perioperative management

### 3.1 Prehospital triage and risk stratification

#### 3.1.1 Clinical scenario

Prehospital triage determines whether severely injured patients reach appropriate trauma centers within critical time windows. Field providers must rapidly assess injury severity from mechanism, vital signs, and anatomic findings to direct patients appropriately. Traditional guideline-based tools demonstrate undertriage rates of 10%–35%, resulting in delayed definitive care and increased mortality (5, 44). The Field Triage Decision Scheme, widely implemented within emergency medical services (EMS), has a sensitivity of only 0.65–0.85 for identifying patients requiring major trauma center resources, with particular deficiencies in geriatric populations where physiologic compensation masks injury severity (32, 33, 44). Meta-analytic evidence indicates that even in well-resourced systems, undertriage rates persistently exceed 10%–15%, directly contributing to preventable deaths (44).

#### 3.1.2 AI technology application

Machine learning algorithms address triage limitations by processing multiple prehospital variables to predict study-specific severe-injury or major-intervention outcomes; for example, one study evaluated major surgery within 12 h after hospital arrival. Random forest and gradient boosting models trained on historical trauma activation datasets of varying scales incorporate multiple input features including vital sign trajectories, injury mechanism descriptors, and demographic characteristics (10, 23, 39).

A nationwide study using the Danish Prehospital Trauma Dataset (*n* = 4,578) evaluated hybrid neural network models for predicting the need for major surgery, achieving Receiver Operating Characteristic (ROC)-AUC values of 0.80–0.86 overall and 0.90–0.95 for major neurosurgery using inputs from the first 5, 10, and 20 min of prehospital contact, though temporally or geographically independent external validation remains pending and performance in subpopulations identifiable by conventional triage criteria may limit incremental clinical utility (10). A prehospital triage model developed from the National Trauma Data Bank achieved an AUC of 0.751 for severe trauma prediction and 0.736 for critical resource use prediction, with a sensitivity of 0.794 and an undertriage rate of 7.8% at a specificity of 0.50, outperforming the Glasgow Coma Scale, Revised Trauma Score, and National Field Triage Guidelines, and was validated using internal validation with 2017–2018 NTDB data and temporal external validation with independent 2019 NTDB data (45, 46).

An alternative critical care transport triage model achieved AUC 0.81 for predicting lifesaving interventions (0.91 for airway interventions), with a specificity of 96.0% but a sensitivity of only 26.8%, illustrating that high discrimination coexisted with low threshold-level sensitivity (39). This model analyzed continuous physiologic waveform signals and vital sign patterns during the first 15 min of patient care, predicting intervention need within 2 min epochs. Among 2,809 trauma patients with 15,088 two-minute epochs, the ensemble machine learning classifier maintained good performance using physiological features captured up to 15 min prior to intervention (39). A prehospital mortality prediction model using only prehospital variables achieved AUC 0.923 with sensitivity 0.780 and specificity 0.880, substantially outperforming shock index (AUC 0.712). External validation across five centers yielded AUCs of 0.895–0.956, although extensive missing predictors in the Australian cohort warrant caution when interpreting international transportability (20).

However, heterogeneity in triage tool operationalization across institutions, including substantial variability in how anatomic and physiologic criteria are defined and measured, complicates model standardization, comparison, and external generalizability (47). Implementation challenges further include algorithmic opacity in deep learning architectures, data quality inconsistencies across emergency medical services systems, and the computational and infrastructure demands of real-time deployment (48, 49).

Clinical Translation Status: Despite promising metrics, clinical adoption of AI triage tools in EMS systems remains limited, with most implementations positioned as secondary decision support rather than autonomous systems, and prospective validation studies remain scarce (11). No AI triage tools have received U.S. Food and Drug Administration (FDA) clearance as standalone devices, with barriers including real-time data integration requirements, liability concerns when AI recommendations conflict with provider judgment, and the need for continuous model updating as populations evolve (23, 24).

### 3.2 Preoperative imaging diagnosis and lesion segmentation

#### 3.2.1 Clinical scenario

Rapid radiological interpretation of whole-body computed tomography scans, comprising 2,000–5,000 images acquired within 3–5 min, represents a critical bottleneck in trauma care. The ever-increasing imaging volume places substantial pressure on emergency and trauma radiologists to provide accurate finalized reports with rapid turnaround times, while the gap between imaging acquisition and formal interpretation creates a risk of delayed recognition of critical findings (36). For pediatric head trauma, AI models have demonstrated capacity to improve diagnostic accuracy for skull fractures and small-volume intracranial hemorrhages, addressing recognized limitations of emergency imaging interpretation in this population (6). The challenge is further compounded by overnight and weekend shifts when radiology coverage is limited, rendering timely finalized reporting operationally difficult and underscoring the need for automated detection support (36).

#### 3.2.2 AI technology application

Deep learning systems employing convolutional neural network architectures perform automated detection and segmentation of traumatic injuries. For intracranial hemorrhage, models process axial CT slices to identify hemorrhage subtypes and quantify volumes, with multiple architectural approaches having been explored across published literature (22). Leveraging spatial relationships across contiguous CT slices enables improved detection of small-volume hemorrhages that may appear ambiguous on individual two-dimensional images (22).

Published AI models for intracranial hemorrhage demonstrate sensitivity of 90%–99% and specificity of 85%–95%, with AUC values of 0.942–0.993 reported across hemorrhage subtypes, with each condition demonstrating at least one operating point at which both sensitivity and specificity exceed 80% [metrics predominantly derived from retrospective cohorts with varying sample sizes and infrequent independent external validation] (6). Real-world evaluation of three commercial fracture detection algorithms (SmartUrgence, Rayvolve, and BoneView) in emergency department patients (*n* = 1,210; 1,500 radiography sets; fracture prevalence 23.7%) demonstrated sensitivity of 90.2%, 92.6%, and 91.3%, and specificity of 92.5%, 70.4%, and 90.5%, respectively, with no significant difference in sensitivity across algorithms (*P* = 0.27); accuracy and specificity were significantly higher for SmartUrgence and BoneView than for Rayvolve (*P* < 0.0001), suggesting that algorithm selection should account for the relative clinical priority of sensitivity vs. specificity; the independence of the radiologic reference standard and repeated examinations within patients require methodological scrutiny (27). AI models demonstrated high accuracy in detecting skull fractures and brain hemorrhage subtypes, with research indicating that AI technologies can reduce diagnostic error rates by a factor of two to three and increase accuracy from 78.1% to 85.2% compared to baseline (6). For solid organ abdominal trauma including spleen, liver, and kidney lesions on CT scans, convolutional neural networks attained over 90% sensitivity and specificity (12). Additional models achieved up to 97% accuracy in diagnosing distal radius fractures on radiographs, 98% sensitivity for hip fractures on pelvic x-rays, and an AUC exceeding 0.80 for intracranial hemorrhage detection on head CT scans (12).

However, performance varies substantially across injury types. Sensitivity decreases substantially for smaller and subacute or chronic hemorrhages, as demonstrated by real-world evaluation showing sensitivity of 45.5% for subacute and 54.8% for chronic intracranial hemorrhage, reflecting the increased complexity of distinguishing subtle pathological findings from normal anatomic variants and imaging artifacts (14, 49). External validation studies reveal performance degradation when models are evaluated on images acquired using CT scanners from different manufacturers or with different acquisition protocols, reflecting the generalization challenges that arise from data heterogeneity, variations in imaging parameters, and differences in patient populations across institutions (49). For lesion segmentation, AI-generated contours compared to expert annotations show variable agreement depending on injury size and type, with smaller lesions such as subcapsular hematomas presenting a greater challenge than larger injuries (22). In commercial ICH accuracy studies, radiology reports used as reference standards and exposure of readers to AI outputs may introduce verification or incorporation bias, as outlined in Supplementary Table S1 and further detailed in the QUADAS-2 assessments in Supplementary Table S2 (13–15). Activation mapping techniques precisely pinpoint 95.9% of hip fracture lesions and models consistently highlight displaced ribs on chest CTs (12).

Clinical Translation Status: Over 15 FDA-cleared AI devices are commercially available for intracranial hemorrhage detection as of 2024, with several achieving Class II 510(k) clearance for computer-aided triage (22, 50). Systems integrate with picture archiving and communication systems to automatically analyze trauma CT scans and send automated critical finding notifications. One single-center implementation study reported that the mean communication time for an ICH finding changed from 70 min before AI implementation to 63 min afterward; report turnaround time for ICH-positive examinations changed from 60 to 63 min (15). In a separate prospective evaluation, mean report turnaround time for ICH-positive examinations was 147.1 min without AI and 149.9 min with AI (*P* = 0.11), with no statistically significant improvement (13). Thus, workflow outcomes differ by measure and setting and should not be generalized across implementations.

However, the impact on clinical outcomes remains uncertain. Retrospective analyses show no significant reduction in time to surgical intervention or mortality in large trauma centers, suggesting imaging interpretation was not the primary rate-limiting step (50, 51). False positive rates generate unnecessary alerts contributing to alert fatigue, a recognized challenge in clinical AI deployment where repeated non-actionable notifications diminish clinician responsiveness to subsequent alerts (22). Limited model interpretability hinders clinician trust, with survey and consensus evidence identifying user acceptance of unexplainable AI outputs and appropriate reliance on AI recommendations as major barriers to clinical deployment (22, 52). Continuous monitoring and retraining requirements as imaging protocols evolve present ongoing operational challenges, with algorithm performance vulnerable to degradation when input data distributions shift due to changes in scanner hardware, acquisition protocols, or patient populations (49).

### 3.3 Perioperative resuscitation and decision support

#### 3.3.1 Clinical scenario

Intraoperative trauma management requires real-time synthesis of evolving physiology, surgical findings, and resource constraints to guide critical decisions including intervention sequencing in polytrauma, resuscitation strategy selection, and determination of damage control vs. definitive repair approaches (53). Massive transfusion protocols employing fixed plasma-to-red blood cell ratios, typically 1:1 or 1:2, fail to accommodate dynamic coagulopathy variability during active hemorrhage, potentially contributing to both under-resuscitation and transfusion complications (30, 40, 41). Decision-making complexity intensifies in damage control laparotomy scenarios where surgeons must balance abbreviated procedures to prevent physiologic exhaustion against the need for definitive repair to avoid repeat operations (54).

#### 3.3.2 AI technology application

Most cited machine learning studies in this subsection predict transfusion requirements or mortality from prehospital or emergency-department information, rather than measuring real-time intraoperative effectiveness. These models predict massive transfusion requirements, defined as 10 or more red blood cell units within 24 h or 5 or more units within 4 h (21). Gradient boosting and random forest algorithms trained on admission vital signs, laboratory values, and injury characteristics enable preemptive massive transfusion protocol activation (21, 55, 56). A multi-algorithm comparison study evaluating K-Nearest Neighbors, Logistic Regression, Decision Tree, Support Vector Machines, Random Forest, XGBoost, and Artificial Neural Networks for predicting triage level, massive transfusion protocol activation, and mortality in 17,390 trauma patients achieved AUC 0.89 for triage, AUC 0.95 for massive transfusion prediction, and AUC 0.99 for mortality prediction, with sensitivities of 0.73, 0.82, and 0.90 and F1 scores of 0.75, 0.42, and 0.79 respectively [retrospective, *n* = 17,390, internal split only, no external validation] (56). The F1 score of 0.42 for massive transfusion protocol (MTP) prediction reflects poor precision-recall balance despite a high AUC, attributable in part to the low event rate of 3.8% in this cohort, where correct classification of the non-MTP majority disproportionately inflates ROC performance (56). Under such class imbalance, sensitivity at a defined clinical threshold and false positive burden per true positive identified are more operationally relevant benchmarks than AUC alone (21, 55). Random forest models demonstrated superior performance, with critical hemodynamic variables including shock indices, systolic blood pressure, and mean arterial pressure identified as most predictive features (56).

Traditional scoring tools such as the Assessment of Blood Consumption score and the Trauma Associated Severe Hemorrhage score provide reasonable but imperfect predictive performance for massive transfusion requirements, representing meaningful performance deficits compared to machine learning approaches (55, 57). In a retrospective nationwide ED registry cohort, a 921-feature AdaBoost model and the AdaBoost/LightGBM combination achieved an AUROC of 0.9974 (compared with 0.9972 for XGBoost) using internal held-out testing. However, 878 features were derived from ICD-10 diagnosis codes, and whether those codes were available at the intended initial-triage decision point was not established. Potential predictor-timing information leakage therefore warrants investigation but should not be presented as a demonstrated mechanism. Class imbalance, model selection and the absence of independent geographic or temporal validation further limit interpretation of near-perfect retrospective estimates. Accordingly, these figures should not be used as prospectively established clinical benchmarks without external evaluation (16). For pediatric populations, the shock index pediatric age-adjusted (SIPA) demonstrated higher specificity but lower sensitivity than age-adjusted tachycardia for identifying severe outcomes including the need for blood transfusion, intensive care unit admission, and mortality (58).

Deep learning approaches employ long short-term memory networks and temporal convolutional networks to process time-series vital sign data, generating dynamic mortality risk estimates that are updated every 5–15 min during resuscitation (8). These models theoretically enable proactive intervention before irreversible physiologic deterioration, though prospective validation of this benefit remains limited (8).

Critical limitations include poor calibration when models are deployed beyond their training environments, susceptibility to overfitting in retrospective cohorts, and the absence of prospective external validation in many published studies, even though selected prehospital models have undergone retrospective temporal or geographic external testing (16, 20, 21, 31, 59). Most reported performance derives from retrospective analyses susceptible to overfitting, with training sets representing 80% of available data and test sets only 20%, potentially inflating performance estimates (56). Lack of prospective validation during actual intraoperative deployment where predictions must be generated from incomplete, noisy data streams represents a fundamental gap in evidence (59).

Clinical Translation Status: No commercially available AI-based intraoperative decision support systems specifically designed for trauma surgery are in widespread use (60). Integration barriers include requirements for connections to multiple data sources including anesthesia information management systems, electronic health records, and laboratory systems, the need for low-latency computation during rapidly evolving scenarios, and unclear regulatory pathways for systems influencing high-stakes treatment decisions (54, 61).

Pilot implementations have generally positioned AI in advisory rather than autonomous roles, with integration challenges including alert fatigue from false positives, difficulty explaining model reasoning to facilitate clinician trust, and a lack of feedback mechanisms to improve real-world performance (22, 52, 60).

### 3.4 Postoperative monitoring and early warning

#### 3.4.1 Clinical scenario

Postoperative trauma patients require continuous surveillance for complications including delayed hemorrhage, evolving coagulopathy, compartment syndrome, acute respiratory distress syndrome, and sepsis. Traditional monitoring through intermittent vital signs, periodic laboratory testing, and clinical assessment may fail to detect subtle early deterioration indicators until decompensation advances (8, 17). Failure to rescue, defined as death following treatable complications, represents a recognized source of preventable mortality in postoperative trauma populations, underscoring the need for more sensitive early detection strategies (8).

Existing early warning scores demonstrate limited predictive accuracy in surgical populations. The Modified Early Warning Score and National Early Warning Score achieve moderate discriminative performance for predicting clinical deterioration events, yet these threshold-based rules applied to individual vital signs fail to capture complex temporal patterns and multivariate interactions preceding deterioration (62). For example, a patient may maintain blood pressure within the normal range while exhibiting progressive tachycardia and narrowing pulse pressure, a pattern indicating compensated shock that single-parameter thresholds miss (8).

#### 3.4.2 AI technology application

Machine learning approaches employ ensemble methods and deep learning trained on high-frequency time-series data from bedside monitors, including continuous heart rate, blood pressure, respiratory rate, oxygen saturation, and electrocardiographic waveforms sampled at 1 s to 1 min intervals (17, 63). Random forest and gradient boosting models process engineered features including heart rate variability metrics, blood pressure trend slopes, and respiratory rate coefficient of variation to predict adverse events 2–24 h before clinical recognition (8, 62). Recurrent neural networks and transformer architectures model complex temporal dependencies in multivariate vital sign data, enabling detection of subtle deterioration patterns manifesting as changes in cross-correlations among variables rather than absolute threshold violations (62, 63).

AI-based early warning systems demonstrate AUC 0.80–0.88 for predicting cardiac arrest, unplanned intensive care unit admission, and rapid response team activation within 24 h, outperforming traditional scores by 0.10–0.15 in AUC (62). An algorithm analyzing echocardiogram videos for abnormal cardiac function achieved AUC exceeding 0.90, while another model predicted cardiac arrest and respiratory failure 1–6 h in advance using only vital signs and history, surpassing traditional early warning scores (12). Sepsis models were developed in a Chinese major-trauma ICU cohort (*n* = 244) and externally evaluated in a separate US cohort (*n* = 717): the highest internally reported AUROC was 0.932 (neural network), whereas the highest externally reported AUROC was 0.891 (XGBoost; LightGBM 0.886) (31). As the maxima involved different algorithms, these values should not be interpreted as a paired within-model validation decline. For acute respiratory distress syndrome prediction in perioperative patients, clinical and physiological risk factors identified through machine learning approaches offer improved prognostic stratification compared to conventional scoring systems (64). AI-enhanced electrocardiography has demonstrated the capacity to support earlier detection of arrhythmias, ischemic changes, and conduction abnormalities relevant to perioperative cardiovascular management, though performance varies substantially by task and clinical context (65).

However, prospective interventional trials demonstrating that acting on AI predictions improves patient outcomes remain scarce. A single-center randomized controlled trial of an AI sepsis prediction system reported significant reductions in in-hospital mortality (8.96% vs. 21.3%, *P* = 0.018) and hospital length of stay (10.3 vs. 13.0 days, *P* = 0.042) in the intervention group (62). However, these findings derive from a single center and multicenter prospective replication is lacking, underscoring that promising trial results do not guarantee generalizable clinical benefit. Beyond the question of clinical efficacy, the operational context of AI monitoring also introduces deployment-specific challenges. High false-positive rates can contribute to alert fatigue and reduce clinician responsiveness, posing a recognized implementation challenge (62, 63). In intensive care implementations, alarm fatigue has been documented as a significant concern, whereby repeated exposure to non-actionable alerts progressively erodes clinician responsiveness to warnings (63). Performance varies across patient subgroups, with lower sensitivity in patients with baseline physiologic abnormalities and those receiving vasoactive medications altering normal vital sign relationships (63).

Clinical Translation Status: Several commercial AI monitoring platforms have been deployed in intensive care units, though widespread adoption across hospital systems remains limited (62). Systems function as clinical decision support providing risk scores and alerts rather than automated interventions (62, 63). Implementation challenges include integration with heterogeneous bedside monitoring equipment from multiple manufacturers, need for alert threshold customization to institutional workflows, and a lack of electronic health record interoperability for documenting AI warnings and clinical responses (49, 63).

Regulatory status varies, with some systems receiving FDA Class II clearance for specific prediction tasks while others operate as institutional quality improvement tools exempt from device regulation (8). Clinical workflow integration requires establishment of response protocols specifying actions to take when alerts are generated, assignment of responsibility for alert review and patient assessment, and mechanisms for feedback when alerts are overridden (8, 63). Economic evaluation of AI monitoring systems remains an active area of study, with potential cost savings from prevented adverse events weighed against substantial implementation costs for software licensing, IT infrastructure, and staff training (63). Clinician acceptance surveys indicate moderate to high confidence in AI early warning systems, with persistent concerns regarding liability, workflow disruption, and clinical judgment skill erosion (8, 52). A consolidated summary of representative use cases and commonly reported performance metrics is provided in Table 1. A schematic overview of current and potential AI applications across the emergency trauma perioperative continuum is provided in Figure 3.

**Table 1:** Selected AI applications and verified model-evaluation settings across emergency trauma care.

Domain	AI approach/study design	Study population/sample	Internal validation/performance	Independent external validation/performance	Important limitations and interpretation	Ref.
Prehospital triage	Histogram gradient boosting; retrospective critical-care transport	USA; single air-transport service; 2,809 patients/15,088 two-minute epochs	INTERNAL patient-grouped 5-fold CV; AUROC 0.810, sensitivity 0.268, specificity 0.960 (epoch level); per-patient undertriage 21.3%.	None; no independent site/time validation.	1,197 otherwise eligible cases excluded owing to unavailable signals/registry data; specialist air transport population.	(39)
Prehospital triage	XGBoost (pTEST); NTDB retrospective model	USA NTDB; development 672,309; internal validation 288,134; 2019 test 508,703	TRAINING AUROC 0.755; INTERNAL (2017–2018) AUROC 0.751 for severe trauma.	TEMPORAL EXTERNAL (2019 NTDB): AUROC 0.750; same registry and health system, not geographic external.	NTDB trauma-centre sampling overrepresents serious injury relative to all EMS encounters; external cohort is from a later time window of the same registry.	(45)
Prehospital surgical triage	Hybrid neural networks; Danish national prehospital cohort	Denmark; *n* = 4,578; training 3,206, tuning/validation 915, holdout 457	INTERNAL 10% same-cohort holdout: AUROC 0.80–0.86 (any major surgery); 0.90–0.95 (neurosurgery).	None; no independent time/site dataset.	Predicts surgery within 12 h after hospital arrival, not necessarily intraoperative need; small surgical subgroups and no external evaluation.	(10)
Prehospital mortality	XGBoost + LightGBM + RF ensemble; Korean KTDB	South Korea training/testing *n* = 204,189; Korea external *n* = 8,358; Australia *n* = 3,578	INTERNAL: AUROC 0.923; sensitivity 0.780; specificity 0.880.	TEMPORAL EXTERNAL (four Korean sites, 2023): AUROC 0.925–0.956; GEOGRAPHIC EXTERNAL (Australia): AUROC 0.895.	At Australian site nine of 21 input variables were wholly missing, with six more missing in approximately 75% of records.	(20)
Emergency neuroimaging	Aidoc commercial ICH triage; prospective two-phase evaluation	USA; one academic centre; 9,954 CT exams from 7,371 patients	Not applicable: pretrained proprietary classifier was not developed or internally validated in this study.	EXTERNAL clinical evaluation: AI alone sensitivity 87.8%, specificity 94.3%, accuracy 93.0%.	No improvement in radiologists' diagnostic performance or turnaround time; differential reference adjudication; mixed-cause ICH rather than trauma-specific cases.	(13)
Emergency neuroimaging	Aidoc ICH detection; before/after implementation study	Switzerland; one hospital; 4,450 CT exams (1,433 before, 3,017 after)	Not applicable: pretrained commercial classifier.	EXTERNAL postdeployment accuracy evaluation: sensitivity 87.2%, specificity 93.9%, accuracy 93.0% (post-AI).	Final clinical radiology reports used as ground truth; level-1 trauma excluded; pre/post workflow comparison is not model validation.	(15)
Emergency neuroimaging	Aidoc ICH triage; retrospective clinical audit	USA; 17 facilities of one Emory health system; 101,944 CT exams/74,142 patients	Not applicable: commercial model developed elsewhere.	EXTERNAL clinical accuracy evaluation: sensitivity 82.2%, specificity 97.6%, accuracy 96.6%.	AI output available to report authors; GPT-4o extracted reference labels from reports; uncertain/postoperative cases excluded; not 17 independent health systems.	(14)
Emergency fracture imaging	Three pretrained classifiers: SmartUrgence, Rayvolve, BoneView	France; one ED; 1,210 patients, 1,500 radiograph sets	Not applicable: models pretrained commercially.	EXTERNAL head-to-head test: sensitivity 90.2%, 92.6%, 91.3%; specificity 92.5%, 70.4%, 90.5%, respectively.	Radiologist-defined image reference; independent confirmation/masking incompletely reported; repeat radiograph sets per patient.	(27)
ED mortality prediction	AdaBoost and AdaBoost + LightGBM (joint-best; AUROC 0.9974); also XGBoost; retrospective nationwide registry	South Korea; NEDIS, >400 hospitals; *n* = 6,536,306 ED trauma presentations	INTERNAL random holdout + 5-fold CV: AdaBoost and AdaBoost + LightGBM AUROC 0.9974; XGBoost 0.9972.	None; no separate geographic/temporal external test.	Best model used 921 variables including 878 ICD-10 diagnosis-code inputs; real-time availability unproven, with possible predictor-timing leakage.	(16)
ED resuscitation prediction	Seven ML classifiers including RF, LR, XGBoost, ANN; retrospective	Qatar; single level-I trauma centre; *n* = 17,390	INTERNAL 80:20 train/test and 5-fold CV: AUROC 0.89 (triage), 0.95 (MTP), 0.99 (mortality); MTP F1 0.42.	None; no independent cohort.	MTP activation occurred in only 3.8%; model selection, imputation and oversampling limit transportability; predicts at admission, not necessarily intraoperatively.	(56)
Prehospital transfusion	Soft-voting RF + AdaBoost ensemble; national registry model	South Korea; KTDB 2017–2022 *n* = 214,772; independent 2023 four-centre cohort *n* = 7,568 (after age exclusion)	INTERNAL discovery-cohort testing: AUROC 0.837.	TEMPORAL EXTERNAL (four Korean regional trauma centres within the KTDB network, 2023): AUROC 0.823 in Results/Table 1; abstract states 0.837.	Final model chosen with reference to performance across evaluation stages; external metric inconsistent within publication; no non-Korean evaluation.	(21)
Trauma ICU sepsis	NN, XGBoost and LightGBM; cohort modelling	China derivation *n* = 244 (8:2 internal split); USA dataset external *n* = 717; total *n* = 961	INTERNAL: NN AUROC 0.932 (model development cohort).	GEOGRAPHIC EXTERNAL (USA): XGBoost AUROC 0.891; LightGBM AUROC 0.886.	Internal/external AUROCs refer to different algorithms, not a paired same-model performance drop; very small derivation cohort; excluded death/discharge in first ICU day.	(31)

**Classification of validation.** Same-data random holdout, cross-validation and bootstrapping were classified as internal. Temporal external validation used a later independent time period; geographic external validation used independent centres or countries. For pretrained commercial imaging classifiers, independent real-world testing was termed external clinical evaluation, not new local model development or internal validation.

**Comparability.** Imaging diagnostic accuracy estimates cannot be compared directly with AUCs from prognostic prediction models; Sun et al. (31) report their highest internal and external AUROCs from different algorithms. Yu et al. (21) report conflicting external AUROC values (0.837 in abstract; 0.823 in Results/Table 1); 0.823 is presented here with this qualification.

**Study scope.** The summary now covers 12 trauma-related model-development and/or external clinical-evaluation studies also shown in Supplementary Table S1. Non-trauma-specific ICH evaluations are presented as indirect imaging evidence, with applicability concerns in S1. Wong et al. (66), which evaluated sepsis risk in a general inpatient population, is not a trauma-specific model evaluation.

**Figure 3:**
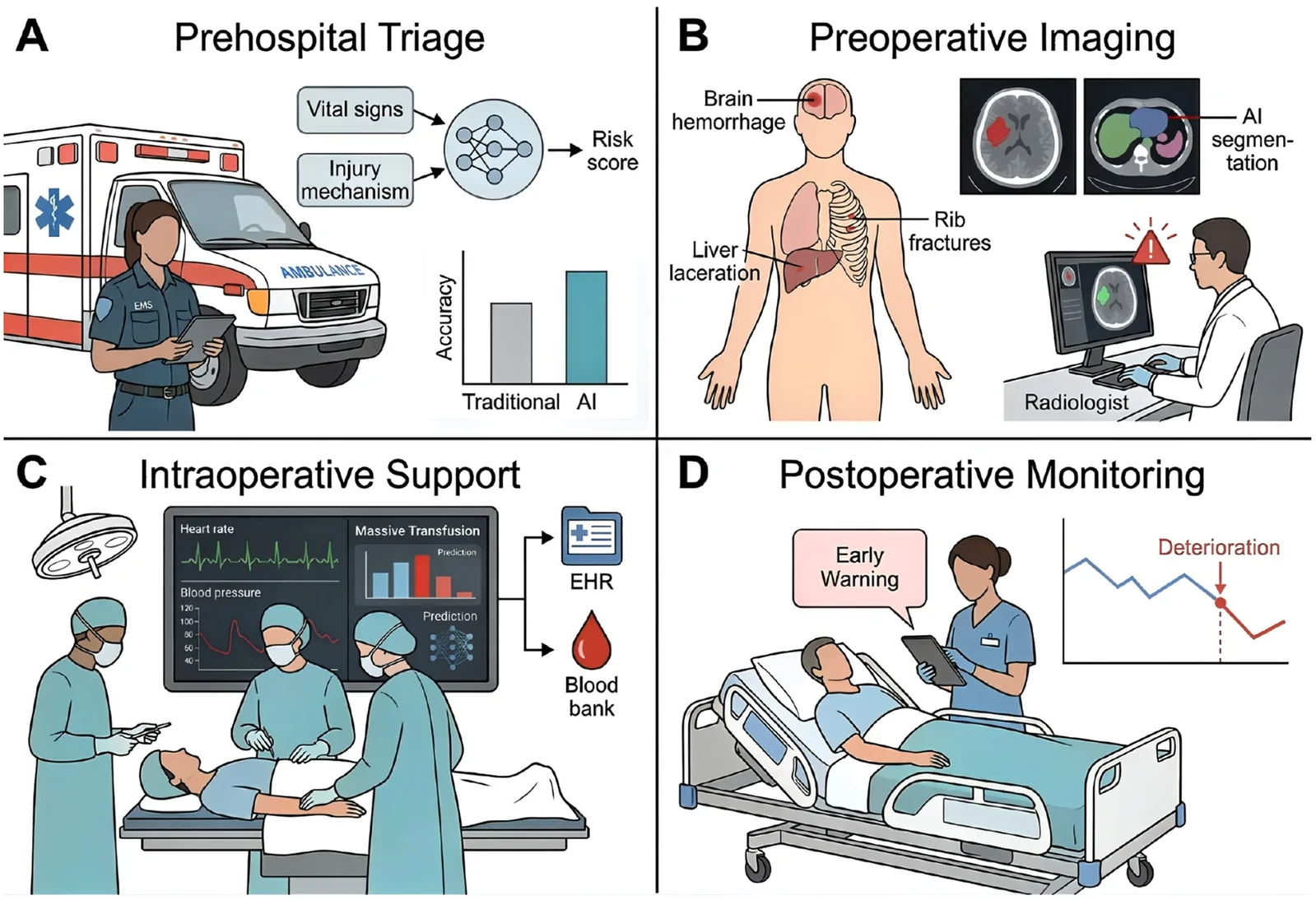
AI applications in emergency trauma perioperative management. **(A)** Prehospital triage using ML-based risk stratification; **(B)** Automated lesion detection/segmentation on CT; **(C)** Intraoperative prediction of transfusion/mortality; **(D)** Postoperative early warning via continuous vital sign analysis. This figure was created by the authors using Figdraw (https://www.figdraw.com; ID: OAWYP5cbbd), an online scientific illustration platform.

## 4 Clinical translation barriers and technical challenges

Despite documented algorithmic performance advantages over conventional methods across multiple trauma care domains, clinical adoption of AI in emergency trauma remains substantially limited due to fundamental implementation challenges (49, 50). External validation studies demonstrate performance degradation when models are deployed beyond their training environments, with heterogeneity in trauma triage tool operationalization across institutions further complicating universal applicability (47–49).

Beyond generalizability limitations, the predominant use of ROC AUC as the primary benchmark represents a specific methodological concern in trauma AI research. In severely class-imbalanced trauma datasets, ROC AUC can remain high even when model performance at clinically relevant thresholds is poor (55, 57). For triage applications, an undertriage rate below 5% represents the operationally defined performance standard in mature trauma systems, yet most published studies do not report model performance at this threshold (44). Precision-recall AUC and decision curve analysis provide more informative assessments under class imbalance and should be reported alongside ROC metrics in future trauma AI studies (55).

The clinical consequences of this metric inflation extend beyond reporting quality and constitute a proximate mechanism of adoption failure. As contextual evidence from general hospitalized patients rather than a trauma cohort, external validation of a proprietary sepsis prediction model found lower-than-anticipated sensitivity and substantial alert burden (66). This pattern is consistent with the well-documented phenomenon of alarm fatigue in intensive care unit (ICU) settings, where sustained exposure to high rates of non-actionable alerts has been shown to progressively condition clinicians to deprioritize warnings, irrespective of whether those alerts are AI-generated or conventional (63, 67, 68). This illustrates why benchmark discrimination must be considered alongside threshold-level performance, prospective workflow effects, and calibration before implementation (69).

Technical and integration barriers include real-time data integration requiring connections to multiple clinical systems, computational latency delaying time-critical decisions, high false positive burdens placing unsustainable demands on clinical workflows, and substantial implementation costs with ongoing maintenance requirements (11, 17, 50, 61).

Ethical and regulatory challenges persist. The black box nature of deep learning limits clinical trust, as reflected in radiologist concerns about understanding AI decision-making (22, 52). Algorithmic bias may perpetuate healthcare disparities, with AI models demonstrating differential performance in geriatric populations where attenuated physiologic responses and comorbidity burden alter variable relationships (32). Regulatory pathways remain unclear, with no FDA-cleared AI prehospital triage tools and unresolved liability frameworks when recommendations conflict with provider judgment (10, 23, 24, 70).

Most critically, return on investment remains uncertain given implementation costs and the lack of demonstrated improvements in patient outcomes. Retrospective analyses show no significant mortality reduction following AI implementation in most domains. While a single-center randomized trial of an AI sepsis prediction system demonstrated statistically significant reductions in mortality and length of stay (62), multicenter prospective confirmation remains absent, suggesting that improved diagnostic accuracy alone does not guarantee system-wide patient outcome gains (50, 63). These barriers must be addressed, along with key technical enablers and clinical translation steps, in order to achieve real-world adoption and outcome improvement.

## 5 Future development directions and recommendations

Technical innovation priorities include multimodal data fusion integrating omics data with clinical phenotypes for precision trauma care, explainable AI architectures providing transparency through attention mapping and saliency visualization, domain adaptation techniques addressing external validation degradation, and generative AI applications for synthesizing training data with strict ethical oversight (22, 48, 52, 71, 72).

Critical research needs are multicenter prospective validation studies with patient outcome endpoints rather than diagnostic accuracy alone, interventional trials evaluating whether acting on AI predictions improves clinical outcomes, subgroup analyses ensuring equitable benefit across diverse populations, and implementation science research examining adoption barriers and optimal human-AI collaboration models (22, 32, 59, 73, 74).

Policy and regulatory frameworks should establish standardized pathways differentiating decision support from autonomous systems, clear accountability and liability boundaries, data governance enabling multi-institutional collaboration while protecting privacy through federated learning, and mandatory algorithmic auditing to identify and mitigate bias (24, 70, 72, 74).

Education and training programs must incorporate AI literacy in medical curricula, critical appraisal skills for practicing clinicians, interdisciplinary training bringing together clinicians and data scientists, and an emphasis on maintaining clinical reasoning to prevent over-reliance on algorithmic outputs (52, 73).

## 6 Conclusion

This narrative review has examined AI applications across the emergency trauma perioperative continuum. A consistent pattern emerges: reported benchmark performance, predominantly derived from retrospective studies under severe class imbalance, may substantially overstate operational utility in many deployment contexts. Systems deployed on the basis of inflated benchmark estimates may generate false positive burdens that risk eroding the clinical responsiveness they were intended to enhance, representing a plausible pathway linking methodological inflation to failed adoption. Based on currently available evidence, a sufficient basis for routine clinical deployment of most AI tools in trauma care has not yet been established. Realizing the genuine potential of AI in this domain will likely require the field to shift from AUC-centered benchmarking toward sensitivity at defined clinical thresholds and patient outcomes in prospective interventional trials. Until performance standards are aligned with operational requirements, the gap between algorithmic promise and clinical reality is likely to persist.
