# Supplementary material for: Artificial intelligence in emergency trauma perioperative management: applications, performance benchmarks, and translation barriers
Source: Front Surg. 2026 Oct 26;13:1970304. doi: 10.3389/fsurg.2026.1970304 (PMC13746039; doi:10.3389/fsurg.2026.1970304)
Supplement: Supplementary file 1 [file Table1.docx]

**Supplementary Table 1. Reassessed risk of bias and applicability using PROBAST domains for trauma-related model performance evaluations**

| **Ref.** | **Study / AI task** | **Model evaluation and validation setting** | **Participants** | **Predictors** | **Outcome** | **Analysis** | **Overall RoB** | **Applicability** | **Domain-specific justification** |
| --- | --- | --- | --- | --- | --- | --- | --- | --- | --- |
| (39) | Weidman et al. \| Prehospital lifesaving-intervention prediction (n=2,809; 15,088 epochs) | Development + INTERNAL evaluation: patient-grouped 5-fold CV and bootstrap resampling; no new-centre test. | High | Low | Low | High | High | High | P: 1,197/4,006 eligible patients excluded because waveform or registry records were unavailable. Pr: signals preceded intervention. O: documented receipt of intervention is measurable, but not equivalent to unmet clinical need. A: incomplete-data selection, tuning/resampling and no reported calibration limit reliability. Applicability: specialist air transport differs from routine field triage. |
| (45) | Chen et al. (pTEST) \| Severe trauma (ISS >=16) and critical-resource prediction | Development n=672,309 + INTERNAL n=288,134 (NTDB 2017-2018); TEMPORAL EXTERNAL n=508,703 (NTDB 2019, same data source). | Low | Low | Low | Low | Low | High | P: defined multicentre cohort of injury patients transported to trauma centres; no case-control sampling reported. Pr: field-available variables. O: ISS and 24-h resource outcomes defined. A: separate internal and temporal test sets, with discrimination, calibration and decision-curve analyses. Applicability (not RoB): NTDB trauma-centre severity mix does not represent all EMS scene encounters; 2019 is not geographically independent. |
| (10) | Millarch et al. \| Prehospital prediction of major surgery (Danish cohort, n=4,578) | Development + INTERNAL only: 70% training, 20% tuning/validation and 10% same-cohort holdout test; no temporal/site external test. | Low | Low | Low | High | High | Low | P: well-defined prehospital trauma cohort. Pr: observed data from the first 5, 10 and 20 min of care. O: major surgery within 12 h of hospital arrival. A: small holdout (~10% of 4,578) and relatively infrequent operation subgroups risk unstable estimates; calibration and optimism correction are insufficiently documented. Absence of external validation alone was not used to assign High RoB. |
| (20) | Oh et al. \| Prehospital ED-mortality prediction (KTDB and Australia) | INTERNAL: KTDB n=204,189 (2017-2022). TEMPORAL EXTERNAL: four Korean sites n=8,358 (2023); GEOGRAPHIC EXTERNAL: Australia n=3,578 (total external n=11,936). | Low | High | Low | High | High | High | P: specified separate trauma datasets. Pr: in the Australian site, 9/21 inputs were wholly absent and six more were missing for ~75% of records, compromising like-for-like measurement. O: death in ED clearly defined. A: high missingness, unclear transportability of imputation/calibration, and post-hoc recalibration require caution. Applicability: Australian input capture materially differs from training. |
| **(13)** | Savage et al. \| Aidoc ICH diagnostic triage (n=9,954 head CT exams) | EXTERNAL clinical evaluation of an existing locked commercial classifier at one centre; prospective two-phase reader comparison; not local model development. | Low | Low | High | Unclear | High | High | P: prospectively assembled adult emergency CT cohort. Pr: locked commercial AI, with undisclosed proprietary development data. O: discordant AI-report pairs received independent expert adjudication (with blinded reviewing where applied), but most concordant pairs were accepted without the same reference process; outcome verification thus differed by AI-report agreement. A: uncertainty remains over verification-related bias in performance estimation. Applicability: ICH was not restricted to trauma. |
| **(15)** | Seyam et al. \| Aidoc ICH detection and ED workflow (n=4,450 CT exams) | EXTERNAL post-deployment clinical evaluation of a pretrained classifier (n=3,017 post-AI CT exams); pre/post workflow comparison is not model validation. | High | Low | High | Low | High | High | P: level-1 trauma CT examinations were excluded, and record identification used ICH-related keywords. Pr: fixed commercial classifier. O: finalized radiology reports served as the reference standard after deployment, without fully independent blinded image adjudication; AI was visible in the clinical workflow. A: detection metrics were reported; major concerns are captured in Participants and Outcome. Applicability: this was a mixed-cause emergency ICH population, not a severe-trauma-only cohort. |
| **(14)** | Chavoshi et al. \| Aidoc ICH detection, real-world retrospective study (n=101,944 CT exams) | EXTERNAL clinical evaluation of an existing commercial model across 17 facilities in ONE health system; proprietary training-data overlap unknown. | High | Low | High | Unclear | High | High | P: postoperative inpatient scans and reports with indeterminate ICH were excluded, which may preferentially remove difficult examinations. Pr: established commercial classifier. O: GPT-4o labels were extracted from clinical radiology reports written with AI results available to readers; even with manually audited report subsets, incorporation and reference-standard error are plausible. A: resampling was by examination rather than patient despite repeat scans. Applicability: mixed-cause ICH within one 17-facility health system. |
| (27) | Bousson et al. \| Three commercial acute-fracture detectors (n=1,210; 1,500 radiograph sets) | EXTERNAL diagnostic accuracy evaluation of THREE existing commercial models at one ED; no local training or internal validation. | Low | Low | Unclear | Unclear | Unclear | Low | P: consecutive ED skeletal-trauma patients. Pr: commercially pretrained fixed algorithms. O: four musculoskeletal radiologists labelled fractures using radiographs; blinding to AI outputs and independent confirmation of occult fractures are not fully established. A: 1,500 sets arose from 1,210 patients, and adjustment for within-patient correlation is not evident. Applicability: acute traumatic fracture detection is directly relevant. |
| (16) | Lee et al. \| ED trauma-mortality prediction, Korean NEDIS (n=6,536,306) | Development + INTERNAL only: five-fold CV and random same-registry holdout; no independent calendar-period or centre-level test. | Unclear | High | Low | High | High | High | P: exclusions and nationwide registry coverage require spectrum review. Pr: AdaBoost and AdaBoost/LightGBM (both AUROC 0.9974) used 921 inputs, including 878 ICD-10 diagnosis-code features; their availability at the moment of intended ED triage was not established. O: ED mortality is objectively defined. A: severe outcome imbalance (0.1%), SMOTE and model selection warrant careful interpretation; information leakage is a concern, not a demonstrated fact. Applicability: full-feature model may not be operable at initial assessment. |
| (56) | El-Menyar et al. \| Trauma triage, MTP activation and mortality (n=17,390) | Development + INTERNAL testing only: 80:20 random split and five-fold CV within one Qatar trauma centre; no external cohort. | Low | Low | Low | High | High | Low | P: defined adult trauma admissions. Pr: admission physiological measurements. O: recorded triage/MTP activation and mortality; activation reflects care delivered, not necessarily blood need. A: imputation and SMOTE/up/down-sampling were used, but fully nested preprocessing and unbiased model selection are insufficiently documented; severe imbalance (MTP 3.8%, F1 0.42). High AUC alone is not evidence of leakage. |
| **(21)** | Yu et al. \| On-scene massive-transfusion prediction in Korean trauma patients | KTDB development + INTERNAL test (2017-2022; n=214,772 after age exclusion); TEMPORAL EXTERNAL evaluation (2023; four Korean regional trauma centres within the KTDB network, n=7,568).. | Low | Low | Low | High | High | Low | P: defined KTDB trauma-registry eligibility and documented age exclusions. Pr: 21 prehospital variables; normalization parameters derived from training data. O: >=5 RBC units in 4 h or >=10 in 24 h was explicitly defined, although transfusion received is a proxy for clinical need. A: the paper states that final model selection drew on performance across all evaluation stages, including external evaluation, compromising a strictly untouched external test. It also gives external AUROC 0.837 in the abstract but 0.823 in the external Results/Table 1. High RoB therefore reflects evaluation/selection concerns, not the mere absence of validation. |
| (31) | Sun et al. \| Sepsis prediction in major-trauma ICU patients (total n=961) | China development n=244, INTERNAL 8:2 split within these 244; USA external dataset n=717 is GEOGRAPHIC EXTERNAL validation (total n=961). | High | Unclear | Unclear | High | High | Unclear | P: excluded death/discharge within first ICU day, imposing survivor selection. Pr/O: admission-time predictors stated, but exact temporal separation from incident sepsis and outcome ascertainment need clarification. A: small internal test set and final algorithm selection using both internal and external results compromise independence; different algorithms top each validation. Applicability: ICU sepsis risk is relevant, but not specifically postoperative care. |

Assessment framework. The four original PROBAST (2019) domains were applied at the level of reported model-performance evaluations, with the development/evaluation distinction clarified using PROBAST+AI (2025). For pretrained diagnostic imaging classifiers, the review additionally considered diagnostic test accuracy principles (patient selection, independent reference standard, verification and masking); a formal QUADAS-2 assessment of these four diagnostic accuracy studies is provided in Supplementary Table S2. The development quality of proprietary systems cannot be inferred from their clinical evaluations. This domain-level review is not a complete formal PROBAST+AI 18-item evaluation.

**Decision rules.** Overall risk of bias (RoB) was High when one or more domains were High; Unclear when at least one domain was Unclear without any High domain; otherwise Low. Overall applicability was evaluated separately against emergency-trauma practice. Missing or insufficiently reported methods were not assumed to be Low risk. The judgments reflect potential bias in reported performance, not the clinical benefit of an AI model.

**Validation and scope.** Internal evaluation denotes development-data resampling or splitting. Temporal external validation uses a separate calendar period; geographic external validation uses distinct centres/countries. An independently tested commercial model is described as external clinical evaluation. The table covers 12 trauma-relevant model evaluations, with non-trauma-specific emergency ICH cases flagged for indirect applicability. Wong et al. (66) was excluded from trauma-specific RoB scoring because its cohort comprised general inpatients.

**Reporting clarifications.** Chen et al. (45) also report an internal validation set (n=288,134). Sun et al. (31) used n=244 for Chinese development/internal evaluation and n=717 for US external evaluation. Yu et al. (21) report 227,567 and 8,867 patients before age exclusion, versus 214,772 and 7,568 afterward; their abstract gives external AUROC 0.837, but external Results/Table 1 give 0.823. Chavoshi et al. (14) is cited as npj Digital Medicine 9:66 (2026).

**Abbreviations.** AUROC, area under the receiver operating characteristic curve; AI, artificial intelligence; CT, computed tomography; CV, cross-validation; ED, emergency department; ICH, intracranial hemorrhage; ICU, intensive care unit; KTDB, Korean Trauma Data Bank; MTP, massive transfusion protocol; NEDIS, National Emergency Department Information System; NTDB, National Trauma Data Bank; RF, random forest; RoB, risk of bias.
