# Supplementary material for: Artificial intelligence in emergency trauma perioperative management: applications, performance benchmarks, and translation barriers
Source: Front Surg. 2026 Oct 26;13:1970304. doi: 10.3389/fsurg.2026.1970304 (PMC13746039; doi:10.3389/fsurg.2026.1970304)
Supplement: Supplementary file 2 [file Table2.docx]

**Supplementary Table 2. Risk of bias and applicability concerns for diagnostic accuracy studies, assessed with QUADAS-2 (n = 4)**

| QUADAS-2 domain | Signalling question / judgement | Savage et al. **(13)** | Chavoshi et al. **(14)** | Seyam et al. **(15)** | Bousson et al. (27) |
| --- | --- | --- | --- | --- | --- |
| Domain 1. Patient selection | | | | | |
|  | Consecutive or random sample of patients enrolled? | Low | Low | High | Low |
|  | Case-control design avoided? | Low | Low | Low | Low |
|  | Inappropriate exclusions avoided? | Low | High | High | Unclear ^d^ |
|  | Risk of bias | Low | High | High | Unclear |
|  | Applicability | High ^a^ | High ^b^ | High ^c^ | Low |
| Domain 2. Index test (AI algorithm) | | | | | |
|  | Index test results interpreted without knowledge of the reference standard? | Low | Low | Low | Low |
|  | If a threshold was used, was it pre-specified? | Low | Low | Low | Low |
|  | Risk of bias | Low | Low | Low | Low |
|  | Applicability | Low | Low | Low | Low |
| Domain 3. Reference standard | | | | | |
|  | Reference standard likely to correctly classify the target condition? | Unclear ^e^ | High ^f^ | High ^g^ | High ^h^ |
|  | Reference standard results interpreted without knowledge of the index test results? | Unclear ^e^ | High ^f^ | High ^g^ | High ^h^ |
|  | Risk of bias | Unclear | High | High | High |
|  | Applicability | Low | Low | Low | Low |
| Domain 4. Flow and timing | | | | | |
|  | Appropriate interval between index test and reference standard? | Low | Low | Low | Low |
|  | All patients received the same reference standard? | High ^i^ | Low | Low | Low |
|  | All patients included in the analysis? | Low | Unclear ^j^ | Unclear ^k^ | Unclear ^l^ |
|  | Risk of bias | High | Unclear | Unclear | Unclear |
|  | Overall risk of bias | High | High | High | High |
|  | Overall applicability concerns | High | High | High | Low |

**Footnotes**

a. ICH was not restricted to trauma; emergency examinations accounted for 55.4% (phase 1) and 57.2% (phase 2) of the cohort, and trauma for 34.8% and 37.3%, respectively.

b. Mixed-cause ICH, including inpatient (20.4%) and outpatient (6.5%) examinations, within a single 17-facility health system.

c. Level-1 trauma examinations excluded by design.

d. Radiography sets lacking the report of any of the three algorithms were excluded (inclusion criterion 4); the number of excluded sets is not reported.

e. Blinded expert review was applied only to AI-report-discordant cases and to a random sample of approximately 10% of concordant cases; the remaining ~90% of concordant cases relied on unverified report-AI agreement.

f. Finalized reports were written with the AI result visible to the reporting radiologist (incorporation bias); reference-standard labels were extracted from these reports by GPT-4o.

g. Finalized reports were written with the AI result visible to the reporting radiologist (incorporation bias).

h. The four musculoskeletal radiologists explicitly used the three AI reports (and follow-up information when available) when establishing the ground-truth diagnosis; no independent CT confirmation of occult fractures was performed.

i. Differential verification: discordant cases and a 10% random concordant sample received blinded expert adjudication; the remaining ~90% did not.

j. Studies not processed by the AI model owing to routine technical issues were excluded, but the number excluded and a flow diagram are not reported.

k. One ICH-negative examination was not analyzed by the algorithm for unknown technical reasons.

l. The number of radiography sets excluded for unavailable algorithm reports is not reported.

Ratings are Low/Unclear/High; superscript letters refer to the footnotes below, and each footnote belongs to the study column in which it is cited. QUADAS-2, Quality Assessment of Diagnostic Accuracy Studies version 2; AI, artificial intelligence; ICH, intracranial hemorrhage; CT, computed tomography. Overall risk of bias was rated High if any domain was rated High, Unclear if no domain was rated High but at least one domain was rated Unclear, and Low otherwise. All four studies also appear in Supplementary Table S1 (PROBAST); this table provides an additional QUADAS-2 assessment because these studies evaluate pre-trained commercial classifiers against a reference standard (diagnostic accuracy) rather than developing or validating a prediction model. Overall applicability ratings are consistent between the two tables. For Bousson et al., QUADAS-2 yields an overall High risk of bias owing to reference-standard concerns, whereas the PROBAST-based assessment in Supplementary Table S1 yields Unclear; this discrepancy reflects differences between the two tools in their handling of reference-standard independence.
